# Some Practical Points in Operating for Cataract

**Published:** 1879-05

**Authors:** De Wecker

**Affiliations:** Paris


### Some Practical Points in Operating for Cataract.

By Dr. DE WECKER, Paris. Translated by Dr. LITTON FORBES, late Clinical Assistant, London Ophthalmic Hospital.

In this lecture, gentlemen, I propose to touch on some points which may arise in your practice, and upon which it is well you should have clear notions. A question, for instance, may present itself as to whether, if one eye only be attacked by cataract, it should be operated on, the sight of the other being good. In answer to this, I would say that no hard and fast rule can be laid down. You must consider the position of the patient in society; whether or no he has to work for his living, and whether or no he has passed his life in a great center of population. If he has to work, and if his business be in a great community—decidedly yes. I should say early, and not wait till he becomes blind. This remark applies with especial force to the cases of those who have to do with machinery, whose indistinct or monocular vision may be a source of extreme danger to themselves or others.

Again, another case: If the cataract of one eye be mature and, that of the other only partially so, what course should be pursued as regards operation? Well, my advice would be, especially with persons in easy circumstance, to operate on the one with as little delay as might be. Many persons are haunted by a nervous dread of blindness, and are especially grateful for the restoration of one eye. Besides, there are other reasons, which I will call moral ones. Many a man dreads above all things to have his correspondence answered or read by others, and the mental annoyance that such a necessity would expose your patient to should be almost sufficient of itself to enable you to decide.

Again, must a cataract be ripe? In my own practice, I look on this as a matter of indifference. I think, however, it is as well to wait until the individual has lost the power of reading with the affected eye before you operate upon it.

If both eyes are cataractous, is it prudent to operate on both at the same time? As a rule, I never hesitate to do so, unless I see some special reason to the contrary. If there be any such—as, for instance, anything not quite normal about the patient's eyes or health, or about your surroundings, or about the course your later operations have taken—then I would say, let a week intervene between your operations.

As a rule, no special preparation of a patient for operation, as used to be done in old days, is at all necssary. I seldom allow patients to lie in bed more than two days, but encourage them to go about as much as possible, taking all due precautions. I have from time to time been compelled to operate upon unfortunates from public institutions, and to send them a distance of eight or nine miles, immediately after the operation, with a single bandage on; nor can I say I have had any bad results from such practice. At the same time, it is one that you adopt only in dire necessity.

I now I come to an important matter; that is, the possible *accidents after operation.* I told you the other day that however skillful you may be as operators, or careful as surgeons, you will assuredly have cases from time to time of suppuration of the cornea and loss of the eye. Now in these cases it is often a question whether the suppuration comes from the iris or the cornea. Does it come from a torn iritis, followed by suppuration, or is it from a keratitis? Jacobson taught a valuable lesson when he recommended the examination of the eye six or eight hours after operation. This was in order that we might see where the mischief really lay, instead of leaving matters during two three days to become worse, as the old surgeons did. While fully agreeing in principle with all that Jacobson has laid down on this subject, I think, nevertheless, that in practice we must not be in too great a hurry to examine the eye lately operated on. I change my bandages after some twelve hours, but, if it seems well, I do not examine the wound at so early a date. Above all, I do not allow the patient to *look down*, for this would tear open the recent wound, and conduce to hemorrhage. I am satisfied with a glance at the vitreous through the pupil, and if that is clear, that is enough.

And now let us state a general proposition, which is, that all accidents of suppuration after cataracts are by *infection*. I am accustomed to describe infection in these cases as of two sorts, namely, *immediate* and *mediate*..

And first as regards immediate infection. This variety does not depend upon the state of health or the constitution of the patient. Anyone is liable to it. It breaks out very quickly, some twelve hours or so after the operation. It invades the cornea, and too often leads to panophthalmitis. Its cause is probably a vitiated conjunctional secretion.

Mediate infection depends much more than the foregoing on the constitutional state of your patient. Its chief exciting cause, however, is a want of coaptation in the corneal wound. Lister's researches have pretty well established that the cause in such cases is the decomposed serosity from the wound, which becomes a source of infection. This want of tone in the tissues, resulting in the failure of coaptation of the lips of the wound, was met by the older surgeons by excessive tight bandaging; but this only made the evil worse, for thereby the various secretions were shut in, instead of being allowed to escape. Mediate infection generally makes its appearance forty-eight hours after operation. It does not cause pain to the patient. It works steadily and, so to say, silently, giving little evidence of its existence. It may, apparently, descend into the eye through the wound, and give rise to an ulcer in the vitreous, from which pus flows out of the eye, while the cornea is perfectly clear. Perhaps this fact may serve to explain a phenomenon which is remarkable enough, namely, that out of the thousands of iridectomies performed, scarcely one ever ends in suppuration; while of cataract operations, however skillfully performed, a certain number always fail from this cause. The reason is, perhaps, because, in the latter the capsule has been opened; moreover, the wound in iridectomy provokes a dispedesis of the vessels in its immediate neighborhood; whereas the wound in the capsule brings the septic poison close to the ciliary body, and into a position from which it is with difficulty eliminated.

The secret of treatment in both these infections, will be found in prophylatic measures. Disinfect your hands, your instruments, the eyes of your patients, especially the infraorbital and supraorbital regions. You have seen me, as a rule, sponging all cases with a weak solution of carbolic acid before operation. This is equally necessary in the case of persons in good society, especially ladies, whose eyelids and brows we constantly find covered with cosmetics and other such filth. You will, of course, cleanse the corneal wound very carefully, and see that the parts are in good apposition, and are not torn open afresh by a too early examination. When iritis supervenes after an operation for cataract, it generally makes its appearance on the fourth or fifth day. At one time the cause of this accident used to be looked for in some bruising the iris had undergone, or in the presence of some cortical masses which had been left behind. The true reason, however, is that a change has taken place in the intraocular pressure, depending most probably on some entanglement of the iris in the wound. At times the iritis or iridochoroiditis takes a neuralgic character. This arises from a mechanical cause, and it is useless to treat it by therapeutics. It can only be cured by mechanical means, namely, by cutting into the wound and freeing the iris, if the exact seat of the entanglement can be found.

Among the operations which have to a certian extent established themselves in ophthalmology as occasionally useful, I would here mention the operation for the removal of the crystalline in its capsule. It is undoubtedly a meritorious operation, and may from time to time be resorted to. I have performed it sixty-six times, and can therefore, perhaps, form a just opinion as to its value. The great objection to this operation is that it scarcely admits of being performed without recourse being had to traction. Now I have already laid down that all methods into which traction entered were radically bad. This operation necessitates it very often, and therefore it stands condemned as a routine procedure. Moreover, be as skillful as you will, you are sure to have a certain number of cases in which the capsule will burst at the precise moment you are extracting the crystalline. Then much of the corticle substance remains behind, which you are obliged to extract with the curette, under less favorable circumstances than if you had used this instrument at first. If some good results have been obtained by the operation, they only, in my mind, go to show how marvelously tolerant the eye is of rough handling.

In conclusion, let me impress on your minds three important facts:

(1.) A good operation for cataract is shown by the easy exit of the lens. The old surgeons allowed it to be driven out of the wound by the mere pressure of the upper eyelid. We cannot quite do that, for our section of the cornea represents a third, instead of a half, of the membrane, as theirs did. Still our close of pressure must be an infinitesimal one, the mere suspicion (loupeon) of a pressure.

(2.) Insure an easy exit of the lens in order to protect yourselves against the iris being strangulated. The more it has been pressed upon, the greater will be the difficulty of saving it.

(3.) As to the infections I have spoken of, all your efforts must be directed to check them at the commencement. For this purpose you must bring to bear all the new facts and discoveries which the modern practice of antiseptic surgery places at your disposal. I am no advocate for performing eye operations under spray. I look forward, however, in the future to our being able very much to diminish the number of suppurations after cataract operation by a more thorough knowledge of antiseptic methods.—*Western Lancet.*

